# New record of the genus *Typhlocolenis* Hoshina, 2008 (Coleoptera, Leiodidae) from South Korea with a key to the species

**DOI:** 10.3897/zookeys.991.55370

**Published:** 2020-11-11

**Authors:** Hideto Hoshina, Sun-Jae Park

**Affiliations:** 1 Faculty of Education, University of Fukui, Fukui City 910-8507, Japan University of Fukui Fukui Japan; 2 National Institute of Biological Resources, Incheon City 22689, South Korea National Institute of Biological Resources Incheon South Korea

**Keywords:** Blind beetle, East Asia, Leiodinae, new species, Pseudoliodini, wingless beetle

## Abstract

This is the first record of the genus *Typhlocolenis* Hoshina, 2008 (Coleoptera, Leiodidae, Leiodinae, Pseudoliodini) in South Korea. Two new Korean species are described, under the names *T. sillaensis***sp. nov.** and *T. jejudoensis***sp. nov.** As a result of this study, the number of *Typhlocolenis* species is now five. A key to the species of the genus is provided.

## Introduction

The genus *Typhlocolenis* belongs to the tribe Pseudoliodini Portevin, 1926 of the subfamily Leiodinae Fleming, 1812 of the family Leiodidae (Perreau 2015) and was established based on three Japanese species by Hoshina (2008). Since then, no species have been added to this genus; therefore, *Typhlocolenis* was considered to be endemic to Japan (Perreau 2015). *Typhlocolenis* is a blind and wingless genus that can be distinguished from the blind genus *Zelodes* Leschen, 2000 of Pseudoliodini by its metaventrite with a median carina. In contrast, *Zelodes* has no median carinae on its metaventrite (Leschen 2000; Hoshina 2008).

In South Korea, three species of two genera of Pseudoliodini, *Dermatohomoeus terrenus* (Hisamatsu, 1985), *Pseudcolenis hilleri* Reitter, 1885, and *P. hoshinai* Park & Ahn, 2007, have been recorded (Park and Ahn 2007; National Institute of Biological Resources 2019). Recently, we studied approximately 20 previously unidentified Korean specimens of Pseudoliodini in the collection of the National Institute of Biological Resources, Incheon. The specimens were collected from forest litter layers by sifting. After careful examination, we discovered that the specimens represented two new species of *Typhlocolenis*. In this paper, we record the genus for the first time in South Korea, describe these new species, and provide a key to the species of the genus.

## Materials and methods

All specimens used in this study were deposited in the National Institute of Biological Resources (**NIBR**), Incheon, South Korea.

The methods were the same as those described in Hoshina (2012). Length and width of head, pronotum, and elytra are measured as follows: length of head is from anterior margin of clypeus to basal margin of head; width of head is between external margins of both eyes; length and width of pronotum and elytra are vertical and horizontal maximum.

### Key to the species of the *Typhlocolenis* genus

**Table:** 

1	Elytra not strigose, bearing large punctures (Fig. 5)	***Typhlocolenis uenoi* Hoshina**
–	Elytra densely and transversely strigose, bearing minute punctures (Fig. 6)	**2**
2	Median lobe of aedeagus broadly rounded at apex in dorsal view (Fig. 12)	***Typhlocolenis furunoi* Hoshina**
–	Median lobe of aedeagus with a nipple at apex in dorsal view (Figs 7, 9, 11)	**3**
3	Median lobe of aedeagus relatively slender and bearing a relatively large nipple at apex in dorsal view (Fig. 11); distribution: Japan (Honshu) (Fig. 15)	***Typhlocolenis fusca* Hoshina**
–	Median lobe of aedeagus relatively thick and bearing a relatively small nipple in dorsal view (Figs 7, 9); distribution: South Korea (Fig. 15)	**4**
4	Median lobe of aedeagus feebly curved at lateral margins in dorsal view (Fig. 7); distribution: mainland of South Korea (Fig. 15)	***Typhlocolenis sillaensis* sp. nov.**
–	Median lobe of aedeagus strongly expanded at about middle of lateral margins in dorsal view (Fig. 9); distribution: Jejudo Island (Fig. 15)	***Typhlocolenis jejudoensis* sp. nov.**

## Taxonomy

### Leiodidae Fleming

#### Leiodinae Fleming

**Pseudoliodini Portevin**

***Typhlocolenis* Hoshina**

##### Typhlocolenis sillaensis

Taxon classificationAnimaliaColeopteraLeiodidae

Hoshina & Park sp. nov.

78C46155-1CB5-5C92-BEF0-B79B7F3485E1

http://zoobank.org/12717A0F-5DAB-4B5C-AE91-0625B08AC153

**Figures 1–6.**
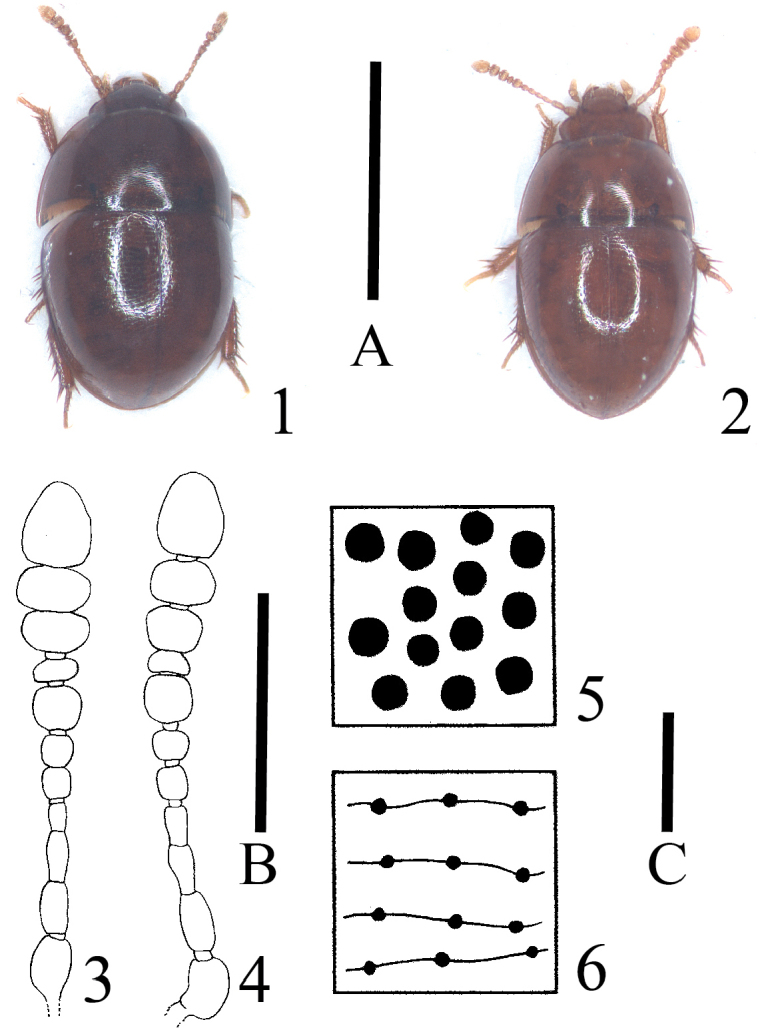
*Typhlocolenis sillaensis* sp. nov. (**1, 3, 6**). *T. jejudoensis* sp. nov. (**2, 4**). *T. uenoi* Hoshina (**5**) **1, 2** habitus (paratypes) **3, 4** antenna **5, 6** elytral punctures. Scale A: 1 mm (**1, 2**). Scale B: 0.2 mm (**3, 4**). Scale C: 0.02 mm (**5, 6**).

**Figures 7–14.**
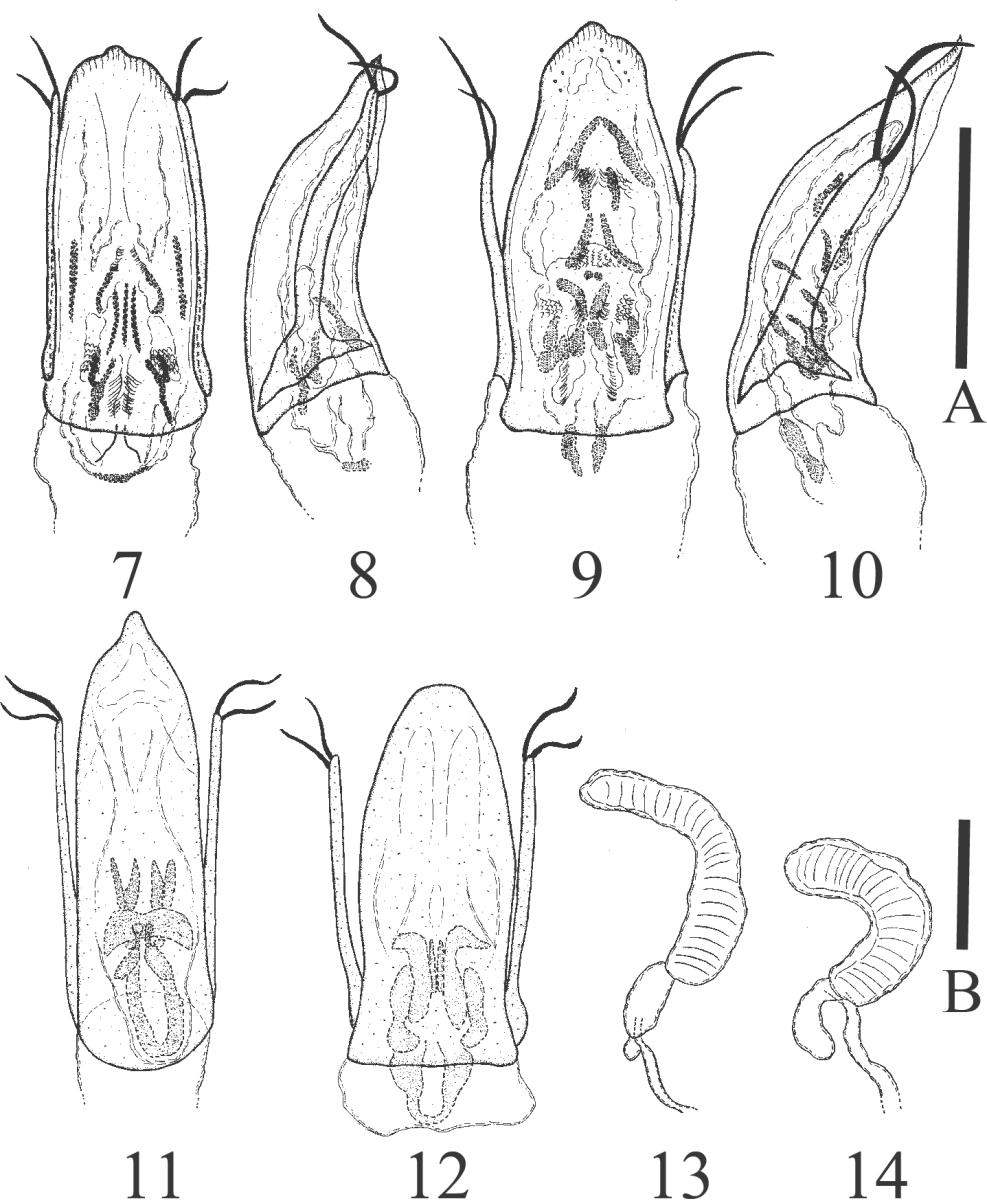
*Typhlocolenis sillaensis* sp. nov. (**7, 8, 13**). *T. jejudoensis* sp. nov. (**9, 10, 14**). *T. fusca* Hoshina (**11**). *T. furunoi* Hoshina (**12**) **7, 9, 11, 12** aedeagus, dorsal view **8, 10** ditto, lateral view **13, 14** spermatheca. Scale A: 0.2 mm (**7–12**). Scale B: 0.05 mm (**13, 14**).

**Figure 15.**
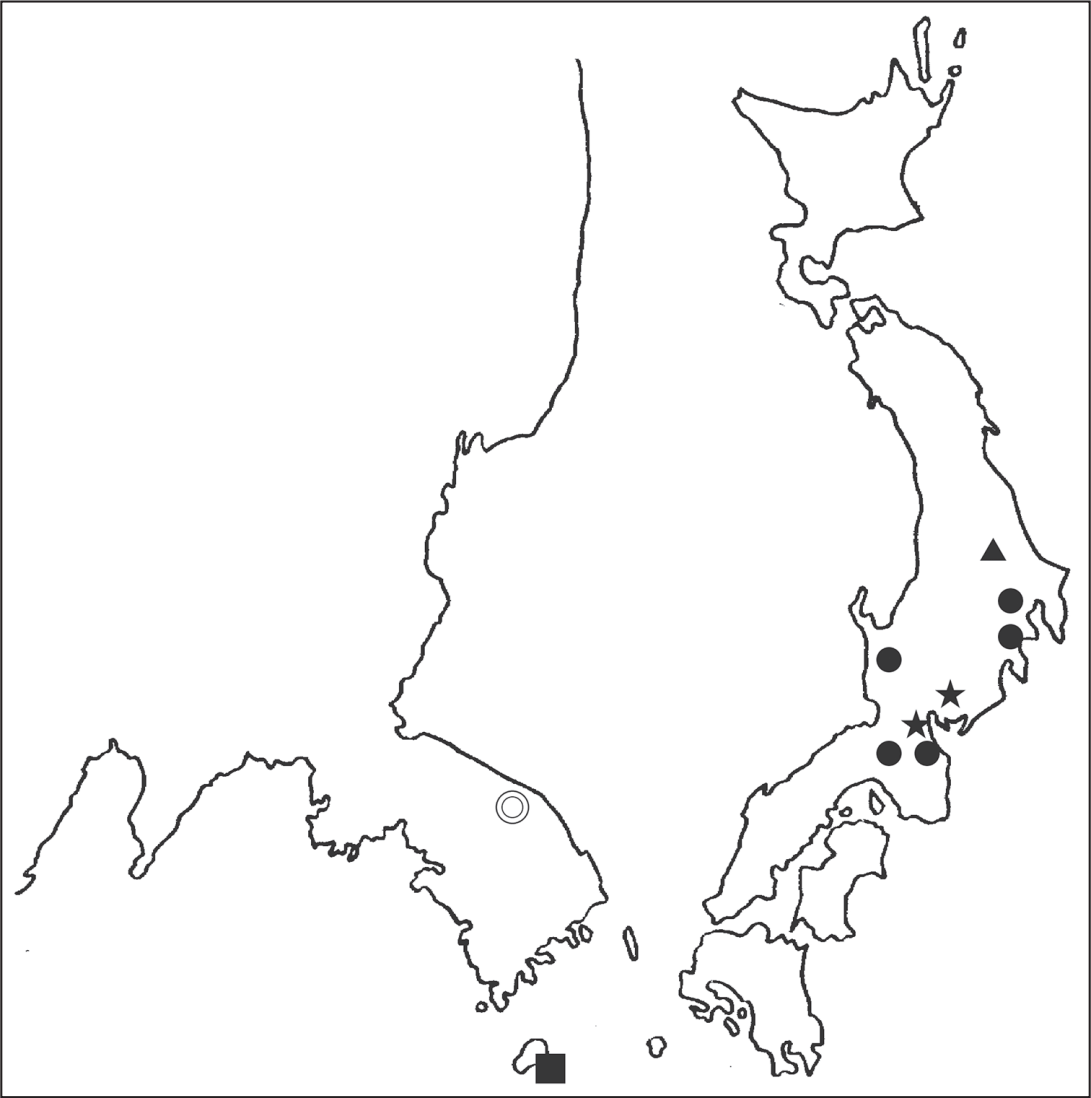
Distribution map of *Typhlocolenis.* Double circle: *Typhlocolenis sillaensis* sp. nov. Square: *T. jejudoensis* sp. nov. Circle: *T. fusca* Hoshina. Star: *T. uenoi* Hoshina. Triangle: *T. furunoi* Hoshina.

Figures 1 , 3 , 6 , 7–8 , 13 , 15

###### Type locality.

South Korea, Gangwon-Prov., Pyeongchang-Gun, Mt. Odaesan.

###### Material examined.

***Holotype***, ♂ (NIBR): KOREA, Gangwon-Prov. Pyeongchang-Gun, Mt. Odaesan, 22. vii. 2004, S.-J. Park leg. ***Paratypes***, 2♂ 1♀ (NIBR): 16. v. 2005, same data as holotype except for the date; 1♂ (NIBR): 21. ix. 2006, same data as holotype except for the date.

###### Diagnosis.

Body length approximately 1.3–1.4 mm. Dorsum almost concolorous, brown, or dark brown. Head and pronotum strongly microreticulate, sparsely and very minutely punctate. Elytra almost smooth, sparsely and very minutely punctate, and densely and transversely strigose. The median lobe of aedeagus relatively thick and feebly curved at lateral margins and bearing a relatively small nipple at apex in dorsal view.

###### Description.

***Measurement of holotype*.** Body length: 1.39 mm; head length: 0.26 mm, width: 0.39 mm; pronotum length: 0.45 mm, width: 0.83 mm; elytron length: 0.81 mm, width: 0.83 mm.

***Coloration*.** Dorsum of body shiny and almost concolorous, brown or dark brown; antennae light brown; mesoventrite brown or dark reddish brown with a black median carina; metaventrite brown or dark reddish brown with a dark brown median carina; abdominal ventrites brown or dark reddish brown; legs brown with light brown tarsi.

Body 1.31–1.39 mm in length, approximately 1.8 times as long as wide (Fig. 1).

Head approximately 1.6 times as wide as long, strongly microreticulate, sparsely and very minutely punctate; head length approximately 0.54 times pronotum length; head width approximately 0.48 times pronotum width; antennomeres 1–4 and 11 longer than wide; antennomere 5–7 almost as long as wide; other antennomeres wider than long; antennomere 11 approximately1.3 times as long as wide (Fig. 3).

Pronotum approximately 1.8 times as wide as long, strongly microreticulate, sparsely and very minutely punctate; pronotum length approximately 0.58 times elytron length; pronotum width almost same as elytron width.

Scutellum weakly microreticulate and almost impunctate or sparsely and very minutely punctate.

Elytra almost as long as wide or slightly wider than long, widest at approximately basal 1/6, almost smooth, sparsely and very minutely punctate, and densely and transversely strigose (Fig. 6).

Legs show no sexual dimorphism on protarsi and normal shape for *Typhlocolenis*.

Meso- and metaventrites strongly microreticulate, almost impunctate, and glabrous; abdominal ventrites strongly microreticulate, almost impunctate, and bearing sparse and very fine pubescences.

Male. Aedeagus generally thick (Figs 7, 8); median lobe of aedeagus feebly curved at lateral margins and bearing a relatively small nipple at apex in dorsal view (Fig. 7) and weakly curved in lateral view (Fig. 8); parameres almost symmetrical and bearing several apical setae (Figs 7, 8).

**Female.** Spermatheca generally crescent shaped (Fig. 13).

###### Etymology.

The specific name is derived from an ancient Korean kingdom, *Silla*, where is the type locality of the present new species.

###### Distribution.

South Korea (Gangwon-Prov.) (Fig. 15).

###### Differential diagnosis.

The genus *Typhlocolenis* is here first recorded in South Korea and is the only blind genus in the Korean Pseudoliodini. Therefore, *T. sillaensis* sp. nov. can be easily separated from *Dermatohomoeus* Hlisnikovský, 1963 and *Pseudcolenis* Reitter, 1885 of the tribe by the lack of eyes. Moreover, *T. sillaensis* sp. nov. can be distinguished from *Dermatohomoeus* and *Pseudcolenis* by having a metaventrite with a distinct median carina. In contrast, *Dermatohomoeus* and *Pseudcolenis* have no median carinae on the metaventrites.

*Typhlocolenis sillaensis* sp. nov. is similar to *T. fusca* Hoshina, 2008 in appearance but can be distinguished by the relatively thick median lobe of aedeagus (Fig. 7) that has a relatively small nipple at the apex in dorsal view (Fig. 7). In contrast, *T. fusca* has a relatively slender median lobe that has a relatively large nipple at the apex in dorsal view (Fig. 11).

###### Natural history.

The life history of *Typhlocolenis sillaensis* Hoshina & Park, sp. nov. is not known.

##### Typhlocolenis jejudoensis

Taxon classificationAnimaliaColeopteraLeiodidae

Hoshina & Park sp. nov.

3AD0A7FE-8078-5897-9215-59806E2A7BC4

http://zoobank.org/D9611B36-5743-4E22-A247-69B106BF4C5F

Figures 2 , 4 , 9 , 10 , 14 , 15

###### Type locality.

South Korea, Jejudo Is., Jeju-City, Goepyeongioreum

###### Material examined.

***Holotype***, ♂ (NIBR): KOREA, Jejudo Is., Jeju-City, Goepyeongioreum, 13. vi. 2005, S.-I. Lee leg. ***Paratypes***, 3♀ (NIBR): same data as holotype; 1♂ 5♀ (NIBR): Jejudo Is., Jeju-City, Bijarium, 12. vi. 2005, S.-J. Park leg.; 2♂ 2♀ (NIBR): Jejudo Is., Jeju-City, Dongbaekdongsan, 13. vi. 2005, S.-I. Lee leg.; 2 exs. (NBIR): Jejudo Is., Jeju-City, Dongbaekdongsan, 22. v. 2006, S.-I. Lee & Y.-H. Kim leg.

###### Diagnosis.

The present species very similar to *Typhlocolenis sillaensis* Hoshina & Park, sp. nov. Body length approximately 1.3–1.4 mm. Dorsum almost concolorous, brown to blackish brown. Head and pronotum strongly microreticulate, sparsely and very minutely punctate. Elytra almost smooth, sparsely and very minutely punctate, and densely and transversely strigose. The median lobe of aedeagus relatively thick, strongly expanded at about middle of lateral margins, and bearing a small nipple at apex in dorsal view.

###### Description.

***Measurement of holotype*.** Body length: 1.30 mm; head length: 0.24 mm, width: 0.35 mm; pronotum length: 0.40 mm, width: 0.71 mm; elytron length: 0.73 mm, width: 0.73 mm.

***Coloration*.** Dorsum of body shiny and almost concolorous, brown to blackish brown; mesoventrite brown or dark reddish brown with a black median carina; metaventrite brown or dark reddish brown with a dark brown median carina; abdominal ventrites brown or dark reddish brown; legs brown with light brown tarsi.

Body 1.28–1.42 mm in length, approximately 1.7 times as long as wide (Fig. 2).

Head approximately 1.5 times as wide as long, strongly microreticulate, sparsely and very minutely punctate; head length approximately 0.61 times pronotum length; head width approximately 0.48 times pronotum width; antennomeres 1–4 and 11 longer than wide; antennomere 5–7 almost as long as wide; other antennomeres wider than long; antennomere 11 approximately 1.3 times as long as wide (Fig. 4).

Pronotum approximately 1.8 times as wide as long, strongly microreticulate, sparsely and very minutely punctate; pronotum length approximately 0.52 times elytron length; pronotum width almost same as or slightly narrower than elytron width.

Scutellum weakly microreticulate and almost impunctate or sparsely and very minutely punctate.

Elytra almost as long as or slightly longer than wide, widest at approximately basal 1/6, almost smooth, sparsely and very minutely punctate, and densely and transversely strigose (Fig. 6).

Legs show no sexual dimorphism on protarsi and normal shape for *Typhlocolenis*.

Meso- and metaventrites strongly microreticulate, almost impunctate, and glabrous; abdominal ventrites strongly microreticulate, almost impunctate, and bearing sparse and very fine pubescence.

**Male.** Aedeagus generally thick (Figs 9, 10); median lobe of aedeagus strongly expanded at about middle of lateral margins, and bearing a small nipple at apex in dorsal view (Fig. 9), slightly, and weakly curved in lateral view (Fig. 10); parameres almost symmetrical and bearing several apical setae (Figs 9, 10).

**Female.** Spermatheca generally C-shaped (Fig. 14).

###### Etymology.

The specific name is derived from the type locality, Jejudo Island.

###### Distribution.

South Korea (Jejudo Island) (Fig. 15).

###### Differential diagnosis.

*Typhlocolenis jejudoensis* sp. nov. is similar to *T. sillaensis* sp. nov. in appearance but can be distinguished by being strongly expanded at about middle of lateral margins in dorsal view (Fig. 9). In contrast, *T. sillaensis* sp. nov. has a very feebly curved median lobe in dorsal view (Fig. 7).

Moreover, *T. jejudoensis* sp. nov. resembles *T. furunoi* Hoshina, 2008 in appearance but can be distinguished by median lobe of aedeagus with a small nipple at the apex in dorsal view (Fig. 9). In contrast, *T. furunoi* has a median lobe broadly rounded at the apex (Fig. 12).

###### Natural history.

The life history of *Typhlocolenis jejudoensis* Hoshina & Park, sp. nov. is not known.

### Notes of the distribution of *Typhlocolenis*

The genus *Typhlocolenis* is distributed only in East Asia (Fig. 15). Among all five species of *Typhlocolenis*, *T. uenoi* Hoshina, 2008 has been collected only in caves (Hoshina 2008). Others have generally been collected in litter layers of forests, although *T. fusca* specimens have sometimes been found in caves (Hoshina 2008). Currently, the two species of *Typhlocolenis* have not been concurrently collected at one location. It is possible that the two Korean species, *T. sillaensis* sp. nov. and *T. jejudoensis* sp. nov. are endemic to the mainland of South Korea and Jejudo Island, respectively (Fig. 15).

## Supplementary Material

XML Treatment for Typhlocolenis sillaensis

XML Treatment for Typhlocolenis jejudoensis
